# Observations of Isolated Mobile Au–Br and Au–S Surface Complexes on Au(100) Electrodes

**DOI:** 10.1002/anie.202520653

**Published:** 2026-02-02

**Authors:** Chaolong Yang, Olaf M. Magnussen

**Affiliations:** ^1^ Institute of Experimental and Applied Physics Kiel University Kiel Germany

**Keywords:** electrochemistry, scanning tunneling microscopy, solid‐liquid interface, surface complexes, surface diffusion

## Abstract

Wet‐chemical processing of metals, for example, galvanic deposition, etching, and nanoparticle synthesis, usually requires complexing agents. In particular, for noble metal processing, anionic complexing species, such as halides or sulfide are indispensable. While it is known that these species strongly adsorb on metal surfaces and affect metal nucleation and growth, the detailed role of these anions in the underlying atomistic processes is less clear. Often, it is assumed that surface complexes are involved, but experimental evidence for the latter is still lacking. Here, we present direct in situ video‐rate scanning tunneling microscopy observations of gold–bromide and gold–sulfur surface complexes on Au(100) electrodes. Based on the intramolecular resolution images obtained in these studies, these species can be assigned to a dimeric planar ${\mathrm{Au}}_{2}{\mathrm{Br}}_{6}$ and a linear ${\mathrm{Au}}_{2}S_{2}$ complex. Once formed, the surfaces complexes are stable even at rather negative potentials and diffuse as molecular species on the Au surface.

## Graphical abstract

Fast scanning tunneling microscopy measurements reveal the formation of Au–Br and Au–S surface complexes on Au(100) electrodes in aqueous electrolytes. These complexes are stable and diffuse as molecular entities over the Au surface, demonstrating that surface transport via complexes has to be considered in wet‐chemical growth and dissolution processes.

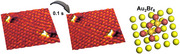

## 1 Introduction

The growth and dissolution of noble metals from liquid phases typically occurs from solutions containing complexes of these metals with strongly bound ligands. In particular, halides, sulfide, and cyanide, which strongly interact with noble metals, are important complexing agents and involved in numerous chemical and electrochemical processes. The presence and chemical nature of these anions strongly influences the growth morphology. Examples include the formation of shape‐controlled nanoparticles [1, 2], as well as homo‐ and hetero‐epitaxial electrodeposition [3, 4, 5, 6]. Understanding the mechanisms that govern the morphological evolution at the metal–liquid interface is still an active field [7]. It specifically requires understanding how these anions affect the attachment and surface transport of the metal species. A key question in that respect is the chemical nature of the adsorbed metal.

In classic treatments of electrodeposition, it is assumed that the deposited metal is attached in form of an adatom, which subsequently migrates over the electrode via surface diffusion, until it is incorporated into the metal lattice [8]. However, because such low coordinated metal adatoms should strongly interact with chemisorbing anions, it is likely that the metal species adsorbs in the form of a surface complex. Evidence for the formation of complexes on metal surfaces has been obtained by scanning tunneling microscopy (STM) and density functional theory (DFT) calculations. Under ultrahigh vacuum (UHV) conditions, Walen et al. identified a set of related Au–S complexes that form on Au(100) [9]. Their results indicate that the ${\mathrm{Au}}_{4}S_{5}$ species, in which the Au adatoms adopt a square arrangement, is the most stable Au sulfide surface complex on Au(100). Another well‐known case is the formation of alkanethiols self‐assembled monolayers (ML) on Au(111), which involves adsorbate complexes incorporating Au adatoms [10, 11]. Furthermore, exposure of CO to Au(111) surfaces was found to induce morphological changes, which were attributed to the formation of mobile Au–CO complexes on the basis of complementary IR spectroscopy and DFT results [12]. However, none of these studies provided direct microscopic observations of migrating surface complexes. The existence of surface complexes during etching and growth at metal–electrolyte interfaces is likewise well‐known [13], but experimental observations up to now were restricted to close‐packed adlayers of Au, Pd, Pt, and Rh halide complexes in which these species were immobile [14, 15, 16, 17, 18, 19]. It is not clear yet, whether these complexes are involved in mass transport on the surface, which governs the evolution of the metal morphology during these processes. Support for an important role of complexes in surface transport comes from DFT studies of Au chloride surface complexes on Au(100). Mesgar et al. reported an adsorbed AuCl species that diffused significantly faster on the surface than Au adatoms [20]. Davila Lopez and Pehlke studied Au diffusion on Au(100) at Cl coverages up to 0.5 ML and found the formation of ${\mathrm{AuCl}}_{2}$ surface species, as well as a Cl vacancy assisted diffusion mechanism [21]. Here, we demonstrate the formation and diffusion of isolated Au–bromide and Au–sulfide surface complexes on Au(100) electrodes, using in situ high‐speed scanning tunneling microscopy (video‐STM). These observations provide experimental evidence for the existence of such species at metal–solution interfaces and show that they can be mobile even in the presence of high‐density anion adlayers.

## 2 Results and Discussion

Video‐STM measurements at 277 K on Au(100) electrodes in solution containing 1 mM KBr show in the potential range 0.05–0.3 $V_{SCE}$, the pseudo‐hexagonal lattice of the commensurate $c\left(\sqrt{2}\times 2\sqrt{2}\right)R45^{\circ }$ Br adlayer structure known from previous surface x‐ray scattering, and STM studies [22, 23]. In this adlayer, the Br adsorbates occupy bridge sites of the unreconstructed Au(100) surface and exhibit spacings of $\sqrt{2}\;d_{Au}$ = 0.408 nm between nearest neighbor adorbates along with the rows parallel to [010] or [001] directions of the Au lattice and between those rows; the spacing to the next‐nearest neighbor Br adsorbates is $\sqrt{5/2}\;d_{Au}$ = 0.456 nm (with Au surface atom spacing $d_{Au}$ = 0.289 nm).

**FIGURE 1:**
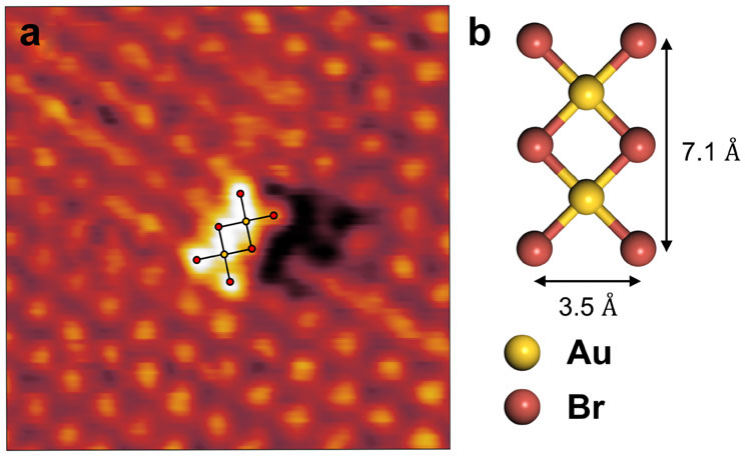
(a) In situ video‐STM image of Au(100) in 1 mM KBr+1 mM ${\mathrm{HClO}}_{4}$ (5 nA tunneling current), showing the rectangular complex formed on the Br adlayer covered surface after increasing the potential to $0.25\;V_{SCE}$. (b) Schematic model of the planar ${\mathrm{Au}}_{2}{\mathrm{Br}}_{6}$ complex. Dimensions are taken from ref. [26].

Upon initial emersion of the sample at potentials ≤0.2 $V_{SCE}$, only the $c\left(\sqrt{2}\times 2\sqrt{2}\right)R45^{\circ }$ Br lattice is observed (see Supporting Information, Figure S1). However, after increasing the potential to $∼0.25\;V_{SCE}$, we observe the formation of additional, larger molecular adsorbate species, displayed in Figure 1a. These species appear higher than the Br adlayer in the STM images and exhibit a rectangular shape. At the corners of these rectangles, distinct maxima are clearly visible, spaced at distances of 0.72 and 0.35 nm, respectively. These distances differ distinctly from the atomic spacings in the $c\left(\sqrt{2}\times 2\sqrt{2}\right)R45^{\circ }$ Br adlayer. Based on their shape and size, the formed molecular adsorbates are assigned to ${\mathrm{Au}}_{2}{\mathrm{Br}}_{6}$, which is the main structural motive in crystalline Au bromide and a stable Au(III) complex in the gas phase [24, 25]. ${\mathrm{Au}}_{2}{\mathrm{Br}}_{6}$ is a trihalide dimer with a planar geometry, in which two gold atoms share two Br ligands, resulting in a four‐fold planar coordination of each Au by Br (Figure 1b). Not only the outer dimensions of the observed adsorbates, but also their appearance in the STM images with two maxima in the molecule center is in good agreement with the reported geometry of ${\mathrm{Au}}_{2}{\mathrm{Br}}_{6}$.

**FIGURE 2:**
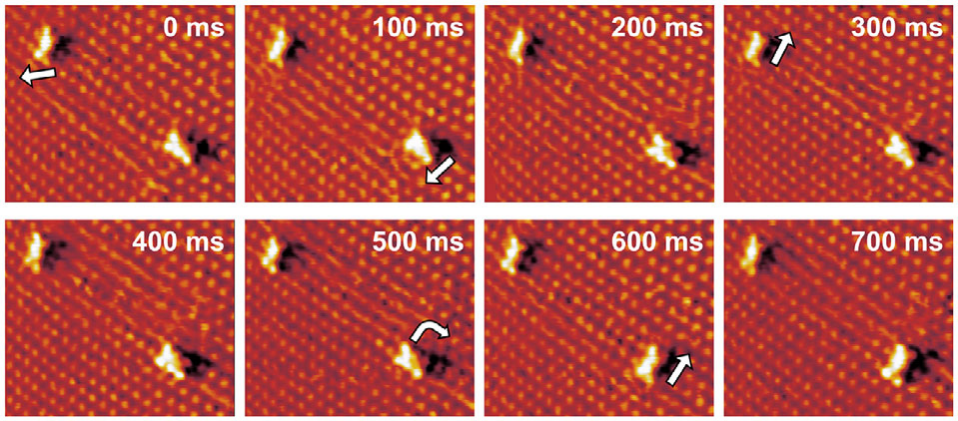
Video sequence of consecutive STM images of Au(100) in 1 mM KBr+1 mM ${\mathrm{HClO}}_{4}$ at $0.25\;V_{SCE}$ (5 nA tunneling current), showing diffusion and rotation of ${\mathrm{Au}}_{2}{\mathrm{Br}}_{6}$ on the Au(100) surface (7.1 nm × 6.1 nm). Motions of the adsorbates are indicated by white arrows. The full video is provided in the supporting information.

Once formed, the ${\mathrm{Au}}_{2}{\mathrm{Br}}_{6}$ species remain stable also at lower potentials, at least within the potential regime of the $c\left(\sqrt{2}\times 2\sqrt{2}\right)R45^{\circ }$ Br adlayer. They exhibit a preferential adsorption orientation along the [010] or [001] directions of the Au lattice. To accommodate these adsorbates, the surrounding Br adlattice is distorted. The latter suggests that the surface complexes adsorb directly on the metal surface, rather than on top of the Br adlayer. Sequences of STM images (Figure 2) reveal diffusion events in which the ${\mathrm{Au}}_{2}{\mathrm{Br}}_{6}$ adsorbates are laterally shifted or rotated by $90^{\circ }$ on the surface. These motions occur via individual jumps in which the entire molecule is displaced, indicating that the ${\mathrm{Au}}_{2}{\mathrm{Br}}_{6}$ adsorbate moves as a molecular species on the metal surface. The typical jump rate is on the order of one‐second in the studied potential regime. A decay of the ${\mathrm{Au}}_{2}{\mathrm{Br}}_{6}$ species into smaller surface complexes or isolated Au adatoms was never observed. In general, other types of Au–bromide complexes were not observed, suggesting that these species, either do not form on the surface or are too short‐lived to be observed by video‐STM. Likewise, ${\mathrm{Au}}_{2}{\mathrm{Br}}_{6}$ species did not attach to each other to form larger complexes. This indicates that ${\mathrm{Au}}_{2}{\mathrm{Br}}_{6}$ is a particular stable surface complex.

The formation of adsorbed ${\mathrm{Au}}_{2}{\mathrm{Br}}_{6}$ is assigned to the onset of oxidative Au dissolution in the form of Au–bromide complexes. Interestingly, earlier in situ STM studies of Au(111) dissolution in Cl‐containing solution reported the formation of long‐range ordered adlayers that were assigned to an assembly of ${\mathrm{Au}}_{2}{\mathrm{Cl}}_{6}$ surface complexes [14]. However, in our case the formation potential of the Au(III) surface complexes ($∼0.25\;V_{SCE}$) is significantly more negative than the Au bulk oxidation potential in Br‐containing electrolyte ($∼0.8\;V_{SCE}$ [27], see also, cyclic voltammograms in Supporting Information, Figure S2). This discrepancy may be explained by oxidation and Au–bromide complex formation at low‐coordinated sites, such as steps and kinks. DFT studies of the Au(100)–Cl system suggest that chlorine could reduce the activation energy for gold atoms detachment from kink sites and result in the formation of Au–Cl complexes [20]. In addition, Masitas et al. reported that the oxidation of small gold nanoparticles occurs at significantly more negative potentials than that of bulk gold in both Br‐ and Cl‐containing solution [27], which likewise can be assigned to the high number of low‐coordinated sites on such particles. Also, for the electrochemical oxidation of Pt, a very similar system, oxidation at steps was found to occur at significantly lower potentials than on the terraces of low‐index surfaces [28]. Video‐STM studies at Au steps (Supporting Information, Figure S3) indeed find strongly dynamic fluctuations near the steps, involving highly dynamic vacancies and adatoms (or complexes) on the upper and lower terrace near the step, respectively. However, we were unsuccessful in clearly observing the formation of a ${\mathrm{Au}}_{2}{\mathrm{Br}}_{6}$ complex at the step. This may be due to the very low oxidation rates at which this process occurs under the employed experimental conditions. For example, in the experiment shown in Figure 2, a ${\mathrm{Au}}_{2}{\mathrm{Br}}_{6}$ coverage of ≈0.003 ML was observed after keeping the potential for 10 min at $0.25\;V_{SCE}$, which corresponds to a formation rate on the order of $5\cdot 10^{-6}$ ML/s. This would not be detectable in electrochemical measurements and result in low probabilities to observe this process in the small surface area probed by video‐STM. On the other hand, an alternative mechanism is conceivable, where the ${\mathrm{Au}}_{2}{\mathrm{Br}}_{6}$ form on the terraces, for example, from the highly mobile Au adatoms that diffuse on the surface after having been detached from steps. More detailed studies will be required for an unambiguous mechanistic understanding of surface complex formation.

**FIGURE 3:**
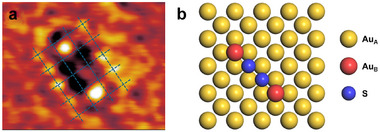
(a) Part of an in situ STM image, showing the Au–S complex on Au(100) in 1 mM ${\mathrm{HClO}}_{4}$ at $0.25\;V_{SCE}$ (9 nA tunneling current). To enhance image quality, 10 consecutive STM frames acquired at 0.1 s intervals were averaged. The Au(100) lattice is illustrated by the dashed blue lines. (b) Schematic model of the proposed Au–S complex on the Au(100) surface.

In a second set of video‐STM experiments, where small amounts of sulfide were dosed on the Au(100) surface (see Supporting Information), a second type of molecular species was found. It appears in the STM images as a linear arrangement of two prominent outer and two weaker inner maxima and was observed in Br‐free perchloric acid solution (Figure 3a), as well as on the $c\left(\sqrt{2}\times 2\sqrt{2}\right)R45^{\circ }$ Br adlayer covered surface (Supporting Information, Figure S4). Once formed, the species remain stable over a potential range of −0.2 to 0.3$\;V_{SCE}$. We attribute them to an adsorbed (anionic) ${\mathrm{Au}}_{2}S_{2}$ surface complex. The ${\mathrm{Au}}_{2}S_{2}^{2-}$ complex is known to be stable in solution, although its precise structure has not been described in the literature [29].

**FIGURE 4:**
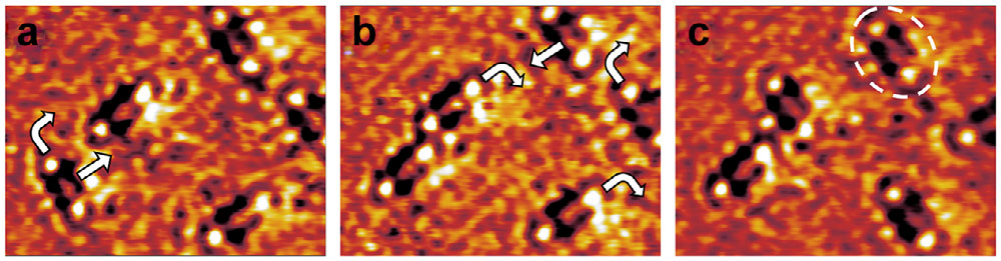
(a)–(c) STM images of Au–S complexes on Au(100) in 1 mM ${\mathrm{HClO}}_{4}$ at $0.25\;V_{SCE}$ (9 nA tunneling current), formed after dosing trace amounts of $S^{2-}$ onto the surface (5.2 nm×4.1 nm). Each image corresponds to the average of five consecutive STM frames acquired at 0.1 s intervals. Translational and rotational motions of the complexes are indicated by white arrows. A complex with slightly tilted inner maxima is highlighted by a dashed ellipse in (c). The full video is provided in the Supporting Information.

Similar to ${\mathrm{Au}}_{2}{\mathrm{Br}}_{6}$, also the ${\mathrm{Au}}_{2}S_{2}$ complex exhibits collective motion, manifesting in lateral translations and $90^{\circ }$ rotations (Figure 4), indicating that also this surface complex moves as a molecular entity over the Au electrode surface. However, in contrast to ${\mathrm{Au}}_{2}{\mathrm{Br}}_{6}$ which shows a preference for lateral translation, ${\mathrm{Au}}_{2}S_{2}$ favors rotation. In addition, the rates at which these diffusion events occur are clearly lower, typically on the order of 10 s. Further insights into the nature of the surface complex can be obtained from a more detailed analysis of the STM images. According to the faintly visible Au lattice and the observed diffusion events, the molecules are oriented parallel to the [011] or [0$\bar{1}$1] directions of the Au lattice. The two outer maxima are always clearly higher than the surrounding Au surface. In addition, extrapolation of the atomic rows of the Au(100) lattice into the area occupied by the molecule (illustrated by blue dashed lines in Figure 3a) indicates that the outer maxima is located in fourfold‐hollow sites on the Au surface. Based on these observations, we assign these maxima to Au adatoms. In contrast, the apparent height in the center of the complex is lower than the surrounding Au surface. We therefore attribute the two weaker central maxima to sulfur atoms, as strongly electronegative adsorbates may even appear as depressions in STM images. This leads to a structure of the ${\mathrm{Au}}_{2}S_{2}$ surface complex, where two Au adatoms, located in Au lattice sites, bind to a bridging disulfide (Figure 3b). In some cases, the two central maxima of the ${\mathrm{Au}}_{2}S_{2}$ complex appear slightly tilted (example highlighted by the dashed ellipse in Figure 4c). Such an arrangement would be expected for covalent Au–S binding, as found in $S_{2}^{2-}$ salts such as pyrite. However, in other observed complexes the weak maxima seem arranged more linearly or exhibit only one clear maximum that typically is located asymmetrically next to one of the Au adatoms. Therefore, further data by other methods are necessary for clarifying in detail the structure of these complexes. In addition, the atoms of the Au substrate lattice next to the complex appear enhanced in the STM images, which most likely is due to electronic effects.

The observed linear ${\mathrm{Au}}_{2}S_{2}$ species, differ from ${\mathrm{Au}}_{4}S_{5}$ reported on Au(100) under UHV conditions [9]. However, no evidence for these or other Au sulfide surface complexes different from ${\mathrm{Au}}_{2}S_{2}$ was found in our studies. This structural difference may be caused by a distinct influence of the electrochemical environment, that is, the presence of solvent molecules, ionic species, and, electric fields at the interface. As in the case of ${\mathrm{Au}}_{2}{\mathrm{Br}}_{6}$, also the formation of the ${\mathrm{Au}}_{2}S_{2}$ complex will involve oxidation of the Au atoms in the complex. Its formation may therefore occur by similar mechanisms as discussed above for ${\mathrm{Au}}_{2}{\mathrm{Br}}_{6}$, apart from the lower surface density and mobility of the complexing agent sulfur. Taken into account the low ${\mathrm{Au}}_{2}S_{2}$ surface diffusion rate, formation on terraces via interaction of sulfur adsorbates, and Au adatoms seems more likely here.

## 3 Conclusion

In summary, our in situ video‐STM studies reveal the formation of stable Au bromide and Au sulfide molecular complexes at electrode surfaces. In contrast to free complexes in solution or the gas phase, where different types of Au bromide and Au sulfide complexes are known, only ${\mathrm{Au}}_{2}{\mathrm{Br}}_{6}$ and ${\mathrm{Au}}_{2}S_{2}$ are observed, indicating that these species are particularly stable on the Au(100) electrode surface. Our studies show that these species are mobile and diffuse over the surface as molecular entities even in the presence of close‐packed anion adlayers. These observations are of substantial relevance for understanding the surface transport mechanisms during Au growth and etching in aqueous solutions. They provide clear experimental evidence that, different from growth under vacuum conditions, nucleation, and growth processes in the presence of these anionic species cannot be described by metal adatom diffusion and attachment/detachment, but require analogue processes based on transport and surface reactions of surface complex species. Obtaining a detailed understanding of the precise mechanisms by which these the surface complexes form and are involved in metal growth and etching processes will be a topic of future research to which our studies open a door. Because surface transport plays a key role in the resulting morphology, such understanding will enable a knowledge‐based development of surface complexation as a tool for process control. These insights will be of relevance for a better understanding and modeling of many applications that employ halide‐ or sulfide‐containing electrolytes, including wet‐chemical and electrochemical Au deposition, Au ore processing, and Au nanoparticle synthesis.

## Conflicts of Interest

The authors declare no conflicts of interest.

## Supporting information


**Supporting File 1**: anie71281‐sup‐0001‐SuppMat.pdf.

**Supporting File 2**: anie71281‐sup‐0002‐VideoS1.wmv.

**Supporting File 3**: anie71281‐sup‐0002‐VideoS2.wmv.

## Data Availability

The data that support the findings of this study are available from the corresponding author upon reasonable request.
