# Supplementary material for: Observations of Isolated Mobile Au–Br and Au–S Surface Complexes on Au(100) Electrodes
Source: Angew Chem Int Ed Engl. 2026 Feb 2;65(11):e20653. doi: 10.1002/anie.202520653 (PMC12970512; doi:10.1002/anie.202520653)
Supplement: Supplementary file 1 — Supporting File 1: anie71281‐sup‐0001‐SuppMat.pdf. [file ANIE-65-e20653-s001.pdf]

Supporting Information for

“Observations of Isolated Mobile Au-Br and Au-S Surface Complexes on Au(100) Electrodes"

Chaolong Yang, Olaf M. Magnussen

1. Experimental details

A home-built electrochemical Video-STM, described in Ref.[1,2], was used to carry out the *in situ* experiments. The temporal resolution is in the range of 0.1 s, which allows direct observation of highly dynamic processes at the interface. The electrochemical cell of the STM is made from Teflon, has a diameter of 8 mm and a height of 3 mm, and is pressed against the Au(100) single electrode surface (Surface Preparation Lab, 10 mm diameter), which forms the bottom of the cell. An Ag/AgCl electrode (eDAQ) or a calibrated platinum wire was used as reference electrode. All potentials are given with respect to the saturated calomel electrode (SCE). A platinum wire was used as counter electrode. Sample and electrochemical cell were kept at a fixed temperature of 277 K by a Peltier element, controlled by a Lakeshore 331 temperature controller. Performing the STM studies at a defined temperature below room temperature allows for more stable imaging and reduces evaporation of the electrolyte. Furthermore, it slows down surface diffusion, which allows better imaging of the mobile surface complexes and helps to exclude the presence of other surface complex species with higher (down to $< 5 \cdot 10^3\, s^{-1}$) diffusion rates.

The following electrolytes were used: 1 mM $HClO_4$ or 1 mM $HClO_4$ + 1 mM KBr prepared from high-purity $HClO_4$ (Merck, suprapure), KBr (Merck, p.a.), and ultrapure water (18.2 MΩcm). The Au(100) single crystal sample was first cleaned by electrochemical oxidation at 4 V in 0.1 M H_2SO_4 for 20 seconds to form a surface oxide layer, followed by immersion in 0.1 M HCl for 4 minutes to remove the oxide. This oxidation–reduction cycle was repeated three to four times. Afterward, the samples were annealed using a butane blowtorch for approximately 5 minutes and then allowed to cool in air. Prior to each preparation step, the crystal was thoroughly rinsed with copious amounts of ultrapure water. For experiments using 1 mM $HClO_4$ as the electrolyte, additional preparation were performed to lift the surface reconstruction of the Au(100). Following annealing and cooling, the sample was immersed in 25 mM HCl for 10 minutes and subsequently rinsed with copious amounts of ultrapure water.

Video-STM data were recorded in constant height mode at image acquisition rates of 10 images per second, using polypropylene-covered tungsten tips. A fixed tunneling bias of +400 mV (sample versus tip) was applied, which ensured that the tip potential remained in the double layer range of the W tip. Tunneling currents between 1 and 15 nA were used, which is sufficient to obtain atomic resolution but still in a range where no acceleration of surface diffusion occurs according to previous studies[3]. On the basis of current-distance spectroscopy measurements on Au(111) electrodes[4], the tip-sample distance at 15 nA should be 5-6 Å larger than that where jump to contact occurs. The sample potential was kept between 0.05 V and 0.3 V (vs SCE). This range is in the potential regime of the $c(\sqrt{2} \times 2\sqrt{2})R45°$ Br adlayer in experiments using 1 mM $HClO_4$+1 mM KBr, and in a potential regime where the Au(100) surface remains unreconstructed in experiments using 1 mM $HClO_4$ alone. To study the Au-S complexes, 2 to 10 μl of a 5 to 10 μM Na_2S (Alfa Aesa, p.a.) was added to the cell (at 0 V vs SCE), resulting in a total amount of S^{2-} in the electrolyte of 10^{-11} to 10^{-10} Mol. After insertion of the sulfide ions about 45 minutes were allowed before approaching the STM tip and starting the measurements. Because of the small depth of the electrolyte layer on top of the sample, this waiting time is enough for the diffusion and irreversible adsorption on the Au surface of all sulfur ions in the electrolyte. On the basis of our previous studies, this procedure leads to S_{ad} coverages of a few percent[3,5].

The presented STM images and videos correspond to the fast current signal channel of the I/V converter, averaged over the time required to scan over the distance of one pixel (see ref.[1,2] for technical details). In this channel, an electronic high-pass filter is employed to improve image quality. This high-pass filtering leads to artifacts in form of black shadows to the left or the right of the adsorbates (depending on scan direction) in the images. For the case of Au_2S_2, performing the experiments at 277 K reduced the number of jumps in an image to 0.5 to 1 s, which allowed improving the image quality by averaging several consecutive images of the video (corresponding effectively to a reduced image acquisition rate). This procedure was used in Figure 3 and 4 of the manuscript (but not in the corresponding attached video).

2. STM video information

Video-Au2Br6: STM video recorded on Au(100) in 1 mM KBr+1 mM $HClO_4$ at 0.25 V_{SCE}, showing diffusion and rotation of Au_2Br_6 on the $c(\sqrt{2} \times 2\sqrt{2})R45°$ Br adlayer covered Au surface (10.2 nm×9.2 nm, 10 Hz image acquisition rate, 5 nA tunneling current).
Video-Au2S2: STM video recorded on Au(100) in 1 mM $HClO_4$ at 0.25 V_{SCE} after dosing trace amounts of S^{2-} onto the surface, showing diffusion and rotation of Au_2S_2 complexes on the Au surface (7.2 nm×8.1 nm, 10 Hz image acquisition rate, 9 nA tunneling current). The abrupt disappearance of one Au_2S_2 adsorbate during the video is a very rare event that most likely is caused by a long-range displacement of this molecule by the STM tip.

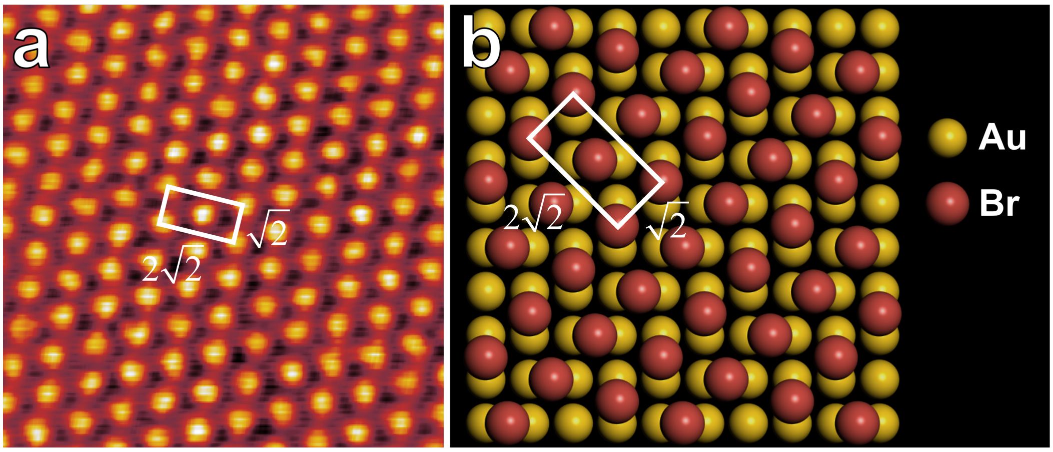

Figure S1. (a) Video-STM image of Au(100) in 1 mM KBr+1 mM $HClO_4$ at $0.1\,V_{SCE}$ (12 nA tunneling current), showing the Br adlayer. (b) Schematic model of the $c(\sqrt{2} \times 2\sqrt{2})R45°$ Br adlayer on Au(100).

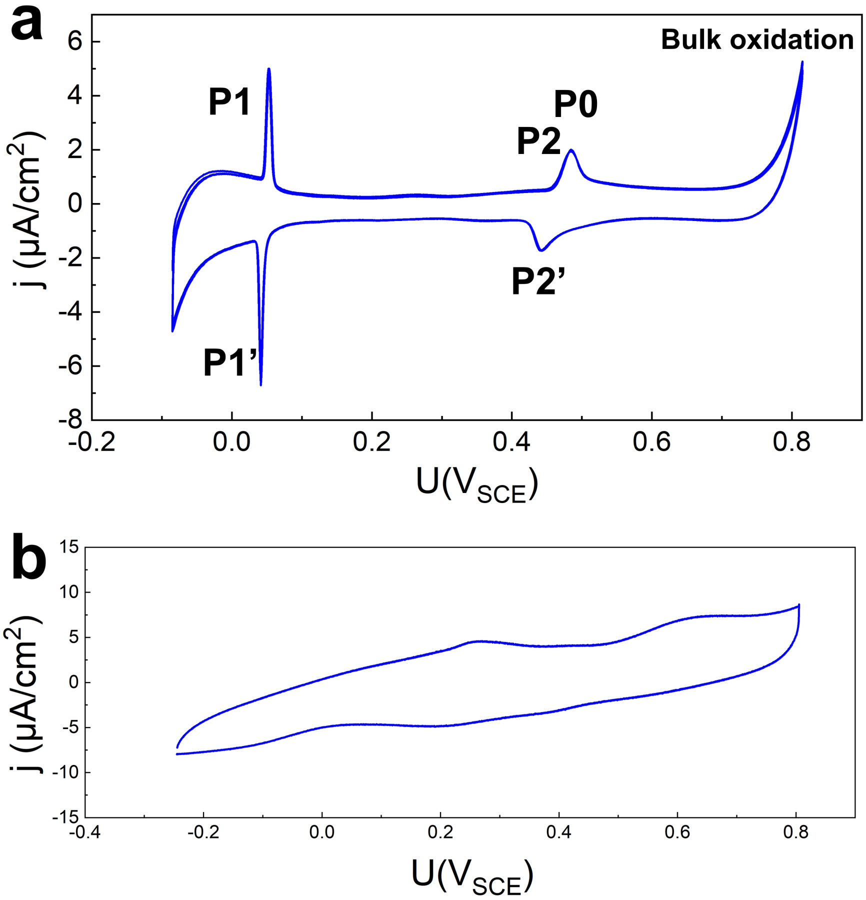

Figure S2. Cyclic voltammograms of the unreconstructed Au(100) single crystal electrode in (a) 1 mM KBr+1 mM $HClO_4$ (10 mV/s) and (b) 1 mM $HClO_4$ (100 mV/s), recorded in the electrochemical cell of the Video-STM at 277 K. Overall, the CVs are in accordance with the literature [6]. The potential region between P1/P1' and P2/P2' in (a) corresponds to the $c(\sqrt{2} \times 2\sqrt{2})R45°$ Br adlayer phase. The CV in pure 1 mM $HClO_4$ solution is largely featureless apart from contributions resulting from residual dissolved oxygen, which cannot be effectively removed from the small STM cell.

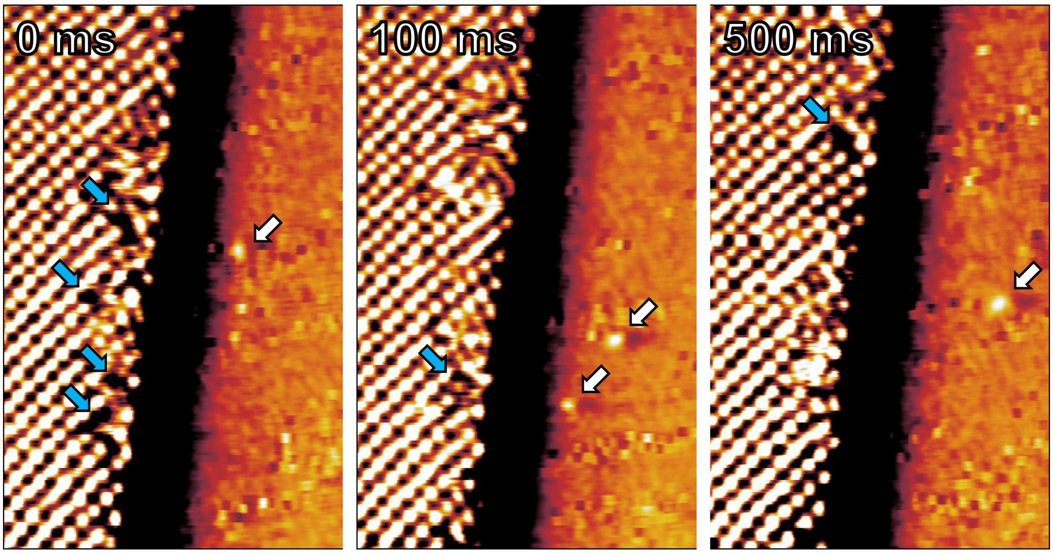

Figure S3. Sequence of Video-STM images recorded on Au(100) in 1mM KBr + 1mM $HClO_4$ at $0.2\,V_{SCE}$ in an area including an Au step (3.4 nA tunneling current). In these constant height mode images, the Br adlayer is only resolved on the upper Au terrace (left hand side), because of the larger tip-sample distance to the lower terrace. The images reveal highly dynamic fluctuations, involving the formation of depressions on the upper terrace and protrusions on the lower terrace of step edges (examples marked by arrows). These features are attributed to Au vacancies (in the upper terrace) and Au adatoms or Au complexes (on the lower terrace) detached from steps, respectively. Their diffusion rates are sufficiently low to allow imaging by video-STM ($< 5 \cdot 10^3\, s^{-1}$), but too high to allow their experimental determination. These observations supports the hypothesis that the Au_2Br_6 complexes are formed from Au atoms detached from steps.

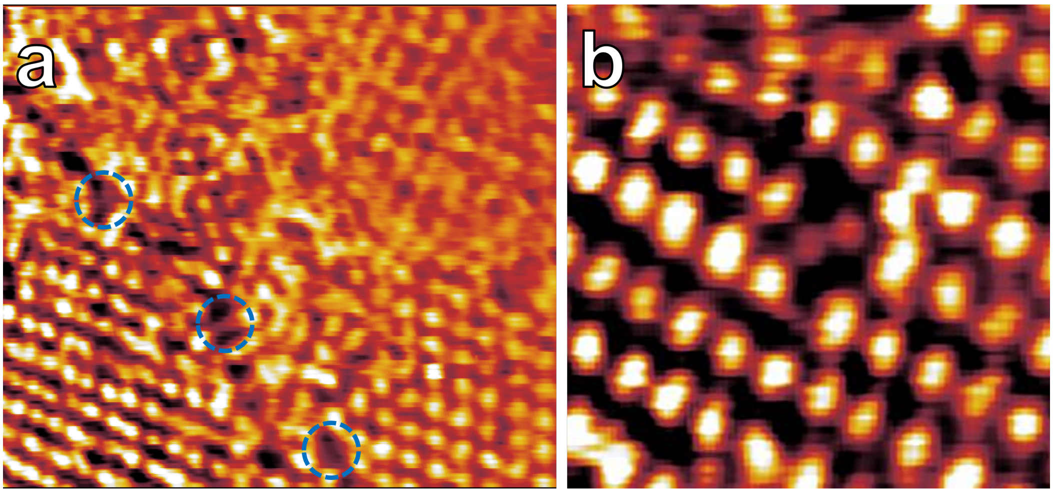

Figure S4. (a) Video-STM image of Au-S complexes on Au(100) in 1 mM KBr+1 mM $HClO_4$ at $0.3\,V_{SCE}$ (3.4 nA tunneling current), formed after dosing trace amounts of S^{2-} onto the surface. The Au-S complexes (highlighted by dashed circles) preferentially accumulate in domain boundaries of the $c(\sqrt{2} \times 2\sqrt{2})R45°$ Br adlayer. (b) Enlarged section of an STM image showing a single Au-S complex in the presence of the $c(\sqrt{2} \times 2\sqrt{2})R45°$ Br adlayer. The Au–S complex induces a local distortion in the surrounding Br adlattice to accommodate it.

References

[1] L. Zitzler, B. Gleich, O. Magnussen, R. Behm, *Proc. Electrochem. Soc.* **2000**, *99-28*, 29.
[2] O. M. Magnussen, *Chem. Eur. J.* **2019**, *25*, 12865.
[3] T. Tansel, A. Taranovskyy, O. Magnussen, *Chem. Phys. Chem* **2010**, *11*, 1438.
[4] M. Hugelmann, W. Schindler, *J. Electrochem. Soc.* **2004**, *151*, E97.
[5] T. Tansel, O. Magnussen, *Phys. Rev. Lett.* **2006**, *96*, 026101/1.
[6] B. Ocko, O. Magnussen, J. Wang, T. Wandlowski, *Phys. Rev. B* **1996**, *53*, R7654.
